# Distinct Vestibular Evoked Myogenic Potentials in Patients With Parkinson Disease and Progressive Supranuclear Palsy

**DOI:** 10.3389/fneur.2020.598763

**Published:** 2021-02-12

**Authors:** Sandra Carpinelli, Philipp O. Valko, Daniel Waldvogel, Elena Buffone, Christian R. Baumann, Dominik Straumann, Esther Werth, Christopher J. Bockisch, Konrad P. Weber, Yulia Valko

**Affiliations:** ^1^Department of Neurology, Clinical Neuroscience Center, University Hospital Zurich, University of Zurich, Zurich, Switzerland; ^2^Sleep & Health Zurich, University Hospital Zurich, University of Zurich, Zurich, Switzerland; ^3^Department of Ophthalmology, University Hospital Zurich, University of Zurich, Zurich, Switzerland; ^4^Department of Otorhinolaryngology, University Hospital Zurich, University of Zurich, Zurich, Switzerland

**Keywords:** vestibular evoked myogenic potentials, oVEMP, cVEMP, Parkinson disease, progressive supranuclear palsy

## Abstract

**Background:** Early brainstem neurodegeneration is common in Parkinson's disease (PD) and progressive supranuclear palsy (PSP). While previous work showed abnormalities in vestibular evoked myogenic potentials (VEMPs) in patients with either disorder as compared to healthy humans, it remains unclear whether ocular and cervical VEMPs differ between PD and PSP patients.

**Methods:** We prospectively included 12 PD and 11 PSP patients, performed ocular and cervical VEMPs, and calculated specific VEMP scores (0 = normal, 12 = most pathological) based on latencies, amplitude, and absent responses. In addition, we assessed disease duration, presence of imbalance, motor asymmetry, and motor disability using the Movement Disorder Society Unified Parkinson's Disease Rating Scale, part III (MDS-UPDRS III). Moreover, we ascertained various sleep parameters by video-polysomnography.

**Results:** PSP and PD patients had similar oVEMP scores (6 [3–6] vs. 3 [1.3–6], *p* = 0.06), but PSP patients had higher cVEMP scores (3 [0–6] vs. 0 [0–2.8], *p* = 0.03) and total VEMP scores (9 [5–12] vs. 4 [2–7.5], *p* = 0.01). Moreover, total VEMP scores >10 were only observed in PSP patients (45%, *p* = 0.01). MDS-UPDRS III correlated with cVEMP scores (rho = 0.77, *p* = 0.01) in PSP, but not in PD. In PD, but not in PSP, polysomnographic markers of disturbed sleep, including decreased rapid eye movement sleep, showed significant correlations with VEMP scores.

**Conclusions:** Our findings suggest that central vestibular pathways are more severely damaged in PSP than in PD, as indicated by higher cervical and total VEMP scores in PSP than PD in a between-groups analysis. Meaningful correlations between VEMPs and motor and non-motor symptoms further encourage its use in neurodegenerative Parkinsonian syndromes.

## Introduction

While widespread brainstem degeneration is common to both Parkinson disease (PD) and progressive supranuclear palsy (PSP), differences in the resulting motor and non-motor phenotypes likely reflect differential anatomical damage by pathological alpha-synuclein in PD and tau proteins in PSP. Substantial clinical overlap especially at disease onset may, however, challenge the distinction between PD and PSP. Ancillary tests, including polysomnography or brain MRI, may reduce this uncertainty by the detection of specific diagnostic clues such as rapid eye movement (REM) sleep behavior disorder (RBD) in PD or selective midbrain atrophy in PSP.

Vestibular evoked myogenic potentials (VEMPs), originally designed as a test of peripheral vestibular function, have emerged as a promising neurophysiologic tool to detect brainstem damage in neurodegenerative and other central neurological disorders (1). VEMPs seem particularly suitable for additional brainstem investigation as they test neuronal circuits extending over the entire brainstem. Cervical VEMPs (cVEMPs) correspond to the vestibulo-collic reflex and depend on the functional integrity of the medial vestibulospinal tract, linking VIII with ipsilateral XI cranial nerve nuclei (2). Ocular VEMPs (oVEMPs), on the other hand, assess the functional integrity of the vestibular pathway underlying the vestibulo-ocular reflex, i.e., the medial longitudinal fasciculus linking VIII with contralateral III cranial nerve nuclei (3).

The discovery that the combined use of oVEMP and cVEMP may reflect brainstem damage in central neurological disorders led to the development of a quantitative VEMP score (4). The VEMP score was originally introduced and validated in patients with multiple sclerosis. It appeared as a reliable tool to detect brainstem involvement and independently correlated with disease disability (4, 5). The total VEMP score represents the sum of four 4-graded scores (0 = normal, 1 = increased latency with normal amplitude and morphology of major potentials, 2 = decrease in amplitude or altered morphology of major potentials, 3 = absence of a major potential), derived from the evaluation of right oVEMP, left oVEMP, right cVEMP, and left cVEMP (5). Minimal and maximal values of the total VEMP score ranges from 0 (normal) to 12 (bilateral absence of potentials in either oVEMP and cVEMP tests).

Several groups reported VEMP abnormalities in PD patients as compared to healthy human subjects (6–9), applying also the above-mentioned VEMP scores (8). Vestibular dysfunction, as assessed by VEMPs, appeared to be more severe in advanced than early PD stages (10). Moreover, numerous correlations between VEMP test results and motor and non-motor symptoms, in particular with postural instability and sleep disturbances (6, 8–11), highlighted the potential role of VEMPs as a meaningful ancillary test in PD. Even in prodromal stages, i.e., in patients with isolated RBD, VEMP scores were shown to be significantly higher than in controls (12, 13). Conversely, the literature on VEMPs in atypical Parkinsonism is scarce, but VEMP abnormalities and an association with increased risk of falling have been reported in PSP patients (11, 14).

Despite this promising emerging evidence for VEMPs in neurodegenerative disorders, a comparative analysis of VEMPs in PD and PSP patients and of their correlations to motor and non-motor clinical signs is still lacking. Hence, the present study assessed frequency and severity of VEMP abnormalities and their differential clinical and polysomnographic correlations in 12 PD and 11 PSP patients, using recently developed and validated oVEMP, cVEMP and total VEMP scores.

## Materials and Methods

The study was conducted at the Department of Neurology, University Hospital Zurich, Switzerland, between October 2017 and January 2019. The Ethics Committee of the Canton of Zurich approved the study protocol (KEK-ZH-Nr 2017-01323). All patients gave written consent prior to study inclusion. The study was carried out in accordance with the Declaration of Helsinki.

### Participants

We prospectively recruited 12 PD patients (4 akinetic-rigid, 2 tremor-dominant, 6 equivalent type) and 21 PSP patients. We excluded patients under antidepressive treatment and with a known vestibular loss or clinically evident hearing impairment, but we did not systematically perform video head impulse testing, audiometry, or tympanometry. Ten of the 21 PSP patients initially consented in participation but then either withdrew during the organizational part of the study or failed to complete all study examinations because they felt overwhelmed by the expected examinations or due to insufficient support by caregivers. Eventually, we included 12 PD and 11 PSP patients in the final analyses. The clinical diagnoses of PD and PSP were made in agreement with recent international criteria and recommendations (15, 16). We assessed motor disability by means of the Movement Disorder Society Unified Parkinson's Disease Rating Scale, part III (MDS-UPDRS III), determined the duration of PD and PSP diseases, and identified the body side with more severe motor symptoms in PD. All PD patients had mild-moderate disease severity as expressed by Hoehn and Yahr scales between 2 and 3. Complaints of imbalance were noted during medical history taking, but no quantitative measures of balance were applied. To assess the potential impact of dopaminergic medications on oVEMP and cVEMP variables, we calculated levodopa equivalent dose (LED) according to published conversion factors (17). Demographic characteristics included age, sex, and body-mass index. We ascertained sleepiness using the Epworth Sleepiness Scale (ESS) (18). Diagnosis of RBD was based on clinical history by bedpartners and polysomnographic findings (19). As a reference for oVEMP and cVEMP analyses, we used normative data from healthy age- and sex-matched subjects from our vestibulo-oculomotor lab.

### Diagnostic Procedures

For the present study, we used the standard oVEMP protocol for testing vestibular function (20). Patients lay with the head supported on a pillow. The skin beneath the eyes and on the chin was cleaned with abrasive skin prepping gel (Nuprep, USA). For each eye, the active electrode (Blue Sensor NF; Ambu, Ballerup, Denmark) was placed beneath the eyes in line with the pupils, the reference electrode directly below the active electrode, and the grounding electrode on the chin. Stimulation with bone-conducted vibration (100 vibration bursts at 500 Hz of 4 ms duration with a repetition rate of 3.1 Hz) was produced by a mini-shaker (4,810, amplifier 2,706; Bruel and Kjaer, Naerum, Denmark) and applied on the forehead during up-gaze. We performed at least two test runs. Recording was done with laboratory data acquisition devices (power 1,401, 1,902 preamplifier; CED, Cambridge Electronic Design, Cambridge, UK). In the averaged recording, we measured the peak-to-peak amplitude n10-p15 and the latency of potentials.

cVEMP were performed in agreement with international guidelines (21). We performed cVEMP in a sitting position using Eclipse hardware platform with VEMP-module (Interacoustics A/S, Audiometer Allé 1, Middelfart, Denmark). The skin over the sternocleidomastoid (SCM) muscles and sternum was cleaned with abrasive skin prepping gel (Nuprep, USA). Active surface electrodes (Blue Sensor NF; Ambu, Ballerup, Denmark) were placed on the belly of the SCM and reference electrodes over the medial clavicle with the ground nearby on the sternum. Patients were asked to turn their head to the side to tense their SCM. Using calibrated headphones (Telephonics TDH-39P; Telephonics Corp., Farmingdale, NY, USA), we applied 200 bursts of air-conducted sound stimuli (500 Hz, 6 ms tone bursts at 90–100 dB normal-hearing level, 100 stimuli) for each side with a repetition rate of 7 Hz. We recorded the background SCM contraction in order to use normalized values of the amplitude instead of the absolute value because the absolute amplitude depends on the level of muscle contraction and is not reliable (22). Therefore, values for cVEMP are unitless. A minimal level of SCM contraction (RMS) of 30 μV was accepted. Bone-conducted cVEMP by vibration was not routinely performed, but was supplemented in those patients in whom air-conducted sound could not elicit any cVEMP signal.

We performed at least two test runs to demonstrate the reproducibility, while responses obtained at the highest stimulus intensity (100 dB NHL, corresponding to 123 dB SPL with our stimulus setup) were considered and averaged. The peak-to-peak amplitude between the first positive and first negative potential (p13-n23) was measured (23). In both oVEMP and cVEMP examinations, we used 0 ms rise times for the stimuli. As reference, we used the data obtained in our lab in healthy subjects, aged 66.1 ± 8.8 and 62.9 ± 13 for cVEMP (*n* = 39) and oVEMP (*n* = 22), respectively.

After performing o/cVEMP, we calculated a total VEMP score (4, 5). As stated above, the VEMP score is based on latency and amplitude of oVEMP and cVEMP. Specifically, in accordance with a previous definition of VEMP scores (4), oVEMP amplitudes were considered abnormal if the amplitude was <0.5 of the mean value of our normative data or when it was decreased >50% compared with the contralateral response. SCM amplitudes were considered abnormal if the amplitude was decreased >1.0 SD compared with the mean value of our normative data or when it was decreased >50% compared with the contralateral response. Latencies were considered prolonged when there was an increase of >2.5 SD of the mean value of our normative data. Minimal and maximal values of the total VEMP score are 0 and 12.

We used a conventional 60-channel polysomnography recording system (RemLogic software; Embla N7000, Embla, Broomfield, CO, USA) and applied a standard montage according to American Academy of Sleep Medicine recommendations (24). Prior to PSG registration, a biocalibration of the eye movements was performed (patients were asked to look up and down then to the left and to the right) to assure the accuracy of the electrooculography (EOG) and also to ensure the preserved ability to gaze upward required for correct oVEMP performance. In all participants, we ascertained a set of standard PSG parameters, including total recording time, total sleep time, sleep onset, sleep efficiency, apnea-hypopnea index, arousal index, frequency of awakenings, and the distribution of distinct sleep stages (N1–3 = non-REM sleep stages 1–3, R = REM sleep stage). Sleep specialists with long-standing experience with sleep examinations in neurodegenerative disorders scored/supervised all polysomnographies.

### Statistical Analysis

We used SPSS 26 (IBM, Armonk, New York, NY, USA) for statistical analyses. We used the Kolmogorov–Smirnov test to determine whether data followed a normal distribution or not. For average comparison of normally distributed data, we used Student's *t*-test. For continuous data that were not normally distributed, we used the Mann–Whitney test. We applied the Kruskal–Wallis test to compare the means of three different PD types. Spearman correlation analyses were used to test for any correlations. For group comparison of nominal data, we used the chi-square test. Significance was accepted at *p* < 0.05.

## Results

Table 1 summarizes the demographic, clinical, and main electrophysiological findings of 12 PD and 11 PSP patients. The two groups did not differ with regard to gender distribution, disease duration, and MDS-UPDRS III, but PSP patients were older than PD patients (71.7 ± 7.9 vs. 62.8 ± 7.1 years, *p* = 0.01). Included PSP patients did not differ from those dropped out with regard to age (74 [64–78] vs. 71 [68–75]; *p* = 0.51), sex (8/11 [73%] vs. 4/10 [40%]; *p* = 0.20), disease duration (4.3 ± 2.9 years vs. 2.6 ± 2.9; *p* = 0.20), and disease disability (34.1 ± 13.4 vs. 31.9 ± 14.9; *p* = 0.69).

**Table 1:** Demographic and clinical characteristics of PD and PSP patients.

	**PD**	**PSP**	***p***
	**(*n* = 12)**	**(*n* = 11)**	
Age, y	64 (58–67)	74 (64–78)	**0.02**
Female gender, *n*	4 (33%)	8 (73%)	0.10
Body mass index, kg/m^2^	24.9 ± 4.1	24.6 ± 3.9	0.87
MDS UPDRS III	23.5 (13.0–36.0)	32.0 (22.8–48.3)	0.07
Duration of the disease, y	7.9 ± 5.5	4.3 ± 2.9	0.07
Levodopa equivalent dose, mg	799 ± 569	463 ± 401	0.12
Balance disturbance, *n*	5 (42%)	11 (100%)	** <0.001**
Epworth sleepiness scale	4.5 (3.0–6.8)	5.5 (3.0–7.0)	0.95
REM sleep behavior disorder	9 (64.3%)	1 (8.3%)	**0.005**
***Polysomnography***	***n=12***	***n=10***	
Total time analyzed (min)	437 ± 35	430 ± 124	0.86
Total sleep time (min)	354 (262–402)	234 (182–295)	**0.007**
Sleep period (min)	421 ± 37	382 ± 118	0.29
Sleep efficiency (%)	85 (64–91)	59 (47–66)	**0.008**
Sleep latency (to first 30s sleep, min)	13 (11-20)	35 (17-91)	**0.02**
Wake after sleep onset (min)	68 (38–143)	137 (111–206)	**0.03**
N1-sleep (% of TST)	22 ± 13	24 ± 27	0.84
N2-sleep (% of TST)	45 ± 9	41 ± 18	0.53
N3-sleep (% of TST)	19 ± 9	25 ± 17	0.29
R-sleep (% of TST)	14 ± 9	10 ± 8	0.25
Arousal index (per hour)	15 ± 5	15 ± 11	0.87
Number of awakenings	22 (12-35)	17 (12-42)	0.66
Apnea and Hypopnea (per hour)	4 ± 4	4 ± 4	0.88
PLMS (per hour)	7 ± 11	23 ± 34	0.14

*Results of parametric t-test are presented as mean ± SD and of non-parametric Mann–Whitney U-test as median (25–75% interquartile range)*.

*MDS-UPDRS, Movement Disorder Society Unified Parkinson's Disease Rating Scale; cVEMP, cervical vestibular evoked myogenic potentials; oVEMP, ocular vestibular evoked myogenic potentials; PLMS, periodic limb movements in sleep syndrome. Bold values indicate statistical significance (p < 0.05)*.

The mean muscular effort maintained by PD and PSP patients during cVEMP testing was similar (PD: 86 ± 21 μV; PSP: 87 ± 48 μV). Two PD patients and 3 PSP patients had low muscular efforts (30–60 μV), with preserved cVEMP signal in both PD and 1/3 PSP patients.

**Figure 1:**
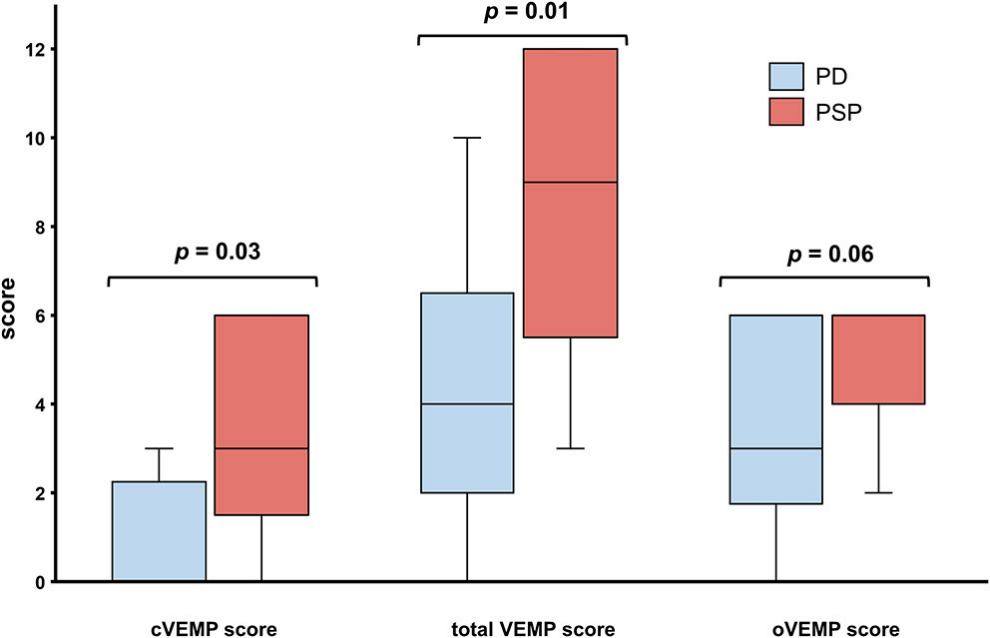
Patients with Parkinson disease (PD, blue) and progressive supranuclear palsy (PSP, red) had similar oVEMP scores, but PSP patients had significantly higher cVEMP and total VEMP scores. Traditional box plots with interquartile range, median (black line), and range (whiskers).

**Figure 2:**
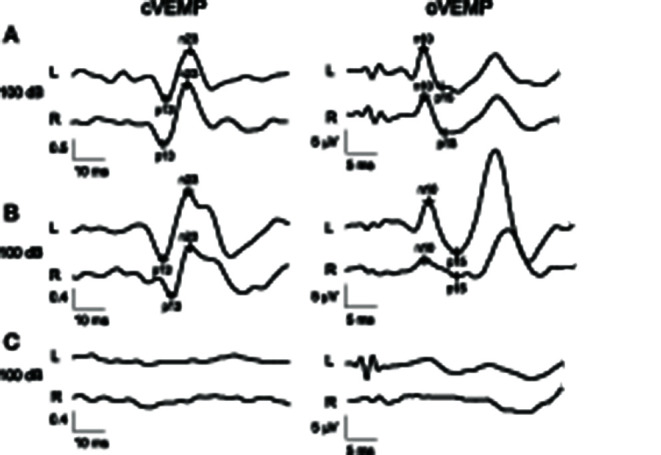
Parkinson disease patient with normal results in both oVEMP and cVEMP and a total VEMP score of 0 **(A)**. Parkinson disease patient with normal cVEMP findings but right-sided oVEMP abnormality with reduced amplitude and a total VEMP score of 2 **(B)**. Patient with progressive supranuclear palsy with absent cVEMP and oVEMP responses on both sides, resulting in a maximal total VEMP score of 12 **(C)**. Values for cVEMP are normalized and therefore unitless. L (left) and R (right) indicate the registration sites.

The various VEMP results are shown in Table 2. In patients with preserved oVEMP and cVEMP signals, latencies, and amplitudes did not show any significant differences. PSP and PD patients had similarly elevated oVEMP scores (6 [3–6] vs. 3 [1.3–6], *p* = 0.06), but the former had significantly higher cVEMP scores (3 [0–6] vs. 0 [0–2.8], *p* = 0.03) and total VEMP scores (9 [5–12] vs. 4 [2–7.5], *p* = 0.01) (Figure 1). Total VEMP scores ≥11 were only seen in PSP patients (*n* = 5, 45%), but not in PD patients (*p* = 0.01). Figures 2A–C provide representative oVEMP and cVEMP recordings of two PD and one PSP patients. In 2 PD and 8 PSP patients, air-conducted sound did not elicit any cVEMP signal; subsequent bone-conducted vibration, however, elicited normal cVEMP signals in both PD patients but only 3/8 PSP patients.

**Table 2:** Ocular and cervical VEMP values in PD and PSP patients.

**VEMP parameters**	**PD**	**PSP**	***p***
**oVEMP**			
n10 latency, ms	13.1 (12.9–13.4)	13.4 (11.9–13.7)	0.60
p15 latency, ms	17.1 (16.4–18.8)	18.4 (16.2–19.5)	0.36
n10/p15 amplitude, μV	7.5 (4.4–9.1)	4.6 (3.4–7.6)	0.30
Absent response, *n*	8/24 (33%)	16/22 (73%)	**0.01**
oVEMP score	3 (1.3–6)	6 (3-6)	0.06
***cVEMP***			
p13 latency, ms	14.0 (13.4–14.3)	14.3 (13.5–15.0)	0.40
n23 latency, ms	22.6 (21.3–23.2)	22.7 (22.0–24.1)	0.54
p13/n23 amplitude	1.2 (0.8–1.6)	0.9 (0.7–1.4)	0.22
Absent response, *n*	4/24 (17%)	12/22 (55%)	**0.01**
cVEMP score	0 (0–2.8)	3 (0–6)	**0.03**
**total VEMP score**	4 (2–7.5)	9 (5-12)	**0.01**

*For each VEMP, data obtained from left and right stimulations have been put together and calculated as one. Median (25–75% interquartile range) values were obtained from patients with preserved VEMPs. Bold values indicate statistical significance (p < 0.05)*.

In either group, age did not correlate with any of the oVEMP and cVEMP measures nor were there any gender-related differences. In both groups, levodopa equivalent doses did not correlate with any of the VEMP scores. There were no differences in any of the VEMP scores between distinct PD disease types (akinetic-rigid, tremor-dominant, equivalent).

In PD patients with asymmetric motor disability, there was no left-right difference in the various oVEMP and cVEMP latencies (Supplementary Table).

In PSP patients, MDS-UPDRS III scores positively correlated with cVEMP scores (rho = 0.77, *p* = 0.01) and higher total VEMP scores (rho = 0.75, *p* = 0.01). In PD patients, however, MDS-UPDRS III did not correlate with any of the VEMP scores. Disease duration did not correlate with any VEMP parameters in either of the two diseases.

Five of the 12 PD patients complained about imbalance, including all 4 patients with akinetic-rigid type, only one with equivalent type and none with tremor-dominant type. Two patients with and two patients without imbalance had absent oVEMP responses. When comparing the various oVEMP latencies only in those with oVEMP responses, imbalance was associated with either significantly or a tendency to delayed latencies (Table 3). Presence or absence of imbalance complaints was not associated with differences in oVEMP amplitudes or with any of the cVEMP parameters.

**Table 3:** Comparison of oVEMP latencies in PD patients with (*n* = 5) and without (*n* = 7) disturbed balance.

	**Complaint of imbalance (*n* = 3)**	**No Complaint of imbalance (*n* = 5)**	***p***
n10 R, ms	14.7	12.9 (12.8–13.2)	0.10
p15 R, ms	19.4	16.7 (16.0–18.4)	0.05
n10 L, ms	13.4	13.0 (12.8–13.1)	**0.047**
p15 L, ms	17.8	16.5 (16.1–17.0)	0.05

*In each group, two patients had absent oVEMP responses and were therefore not included in the statistical analysis. Bold values indicate statistical significance (p < 0.05)*.

**Figure 3:**
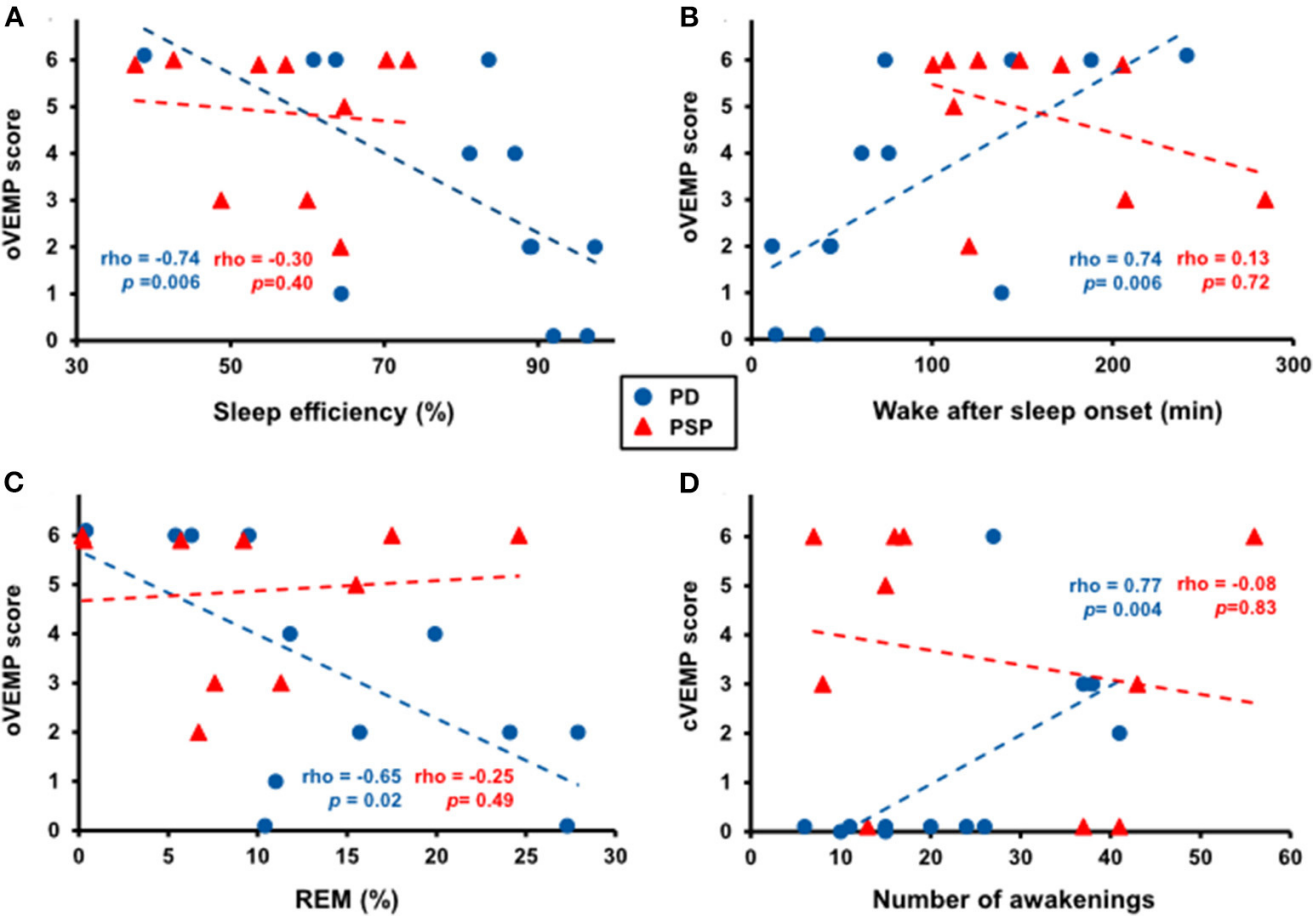
Correlation between several polysomnographic markers of disturbed sleep and VEMP abnormalities in patients with Parkinson disease (PD, blue dots) but not with progressive supranuclear palsy (PSP, red triangles). Higher oVEMP scores significantly correlated with lower sleep efficiency **(A)**, more time spent awake after sleep onset **(B)**, and less rapid eye movement (REM) sleep **(C)** in PD patients. Higher cVEMP scores significantly correlated with more awakenings in PD patients **(D)**.

In PD patients, several polysomnographic markers of disturbed sleep were associated with VEMP abnormalities (Figures 3A–D). Specifically, oVEMP scores correlated negatively with sleep efficiency (rho = −0.74, *p* = 0.006), positively with wake after sleep onset (rho = 0.74, *p* = 0.006), and negatively with R sleep (rho = −0.65, *p* = 0.02). cVEMP scores, on the other hand, correlated positively with the number of awakenings (rho = 0.77, *p* = 0.004). Conversely, the presence of RBD and the ESS score did not correlate with any of the VEMP scores.

## Discussion

Using a recently developed VEMP score, we found in a between-groups analysis significantly higher cVEMP and total VEMP scores in PSP patients than in PD patients. In addition, the severity of VEMP abnormalities correlated with various clinical symptoms, including motor disability in PSP patients, reported imbalance in PD patients, and nocturnal sleep problems as documented by video-polysomnography in PD patients. Very high total VEMP scores (11 and 12) were only seen in PSP patients (36%).

In the absence of other comparative VEMP analyses between PD and PSP, the observed significant difference in VEMP abnormalities and its potential diagnostic implications need to be replicated by other groups. A recent study compared VEMP abnormalities in PD and multiple system atrophy, two alpha-synucleinopathies, and failed to detect any differences (25). The observation of more pronounced VEMP abnormalities in PSP than in PD patients suggests that tau pathology in PSP more severely damages the vestibular nuclei and their neural pathways in the brainstem than the alpha-synuclein pathology does in PD.

The observed difference in cVEMP abnormalities between PSP and PD patients may also serve as a sensitive neurophysiologic reflection of postural instability and risk of falls, which is a diagnostic hallmark in PSP and typically more pronounced than in PD. It is in line with an earlier study of Liao et al., which reported reduced cVEMP amplitudes in 10 PSP patients compared to 30 controls, and suggested that impaired vestibulo-spinal reflexes contributed to postural instability in PSP (14). While Liao et al. also measured reduced oVEMP amplitudes in PSP, other groups failed to confirm VEMP abnormalities in PSP (26, 27). Moreover, although cVEMP and oVEMP scores did not differ in PD patients with and without a complaint of imbalance, we found delayed oVEMP latencies in those with imbalance. A significant association between VEMP abnormalities and postural instability in patients with PD has been previously reported, using various measures of postural instability such as the Mini-BEST score (8) or the clinically tested risk of falling (11). Imbalance and impaired postural reflexes in PD and PSP are likely multifactorial, and our findings suggest that neurodegenerative damage of central vestibular pathways contributes to this salient clinical sign. Indeed, neuropathological studies showed accumulation of pathological proteins and neuronal loss in vestibular nuclei in PD (28, 29), as well as in PSP (30).

Sleep-wake disturbances are a prominent non-motor symptom in both PD and PSP. Based on questionnaires only, previous studies found that the extent of VEMP abnormalities correlated with sleep problems, daytime sleepiness, fatigue, and the presence of RBD (8–10). Our study extends this association by showing that various polysomnographic measures of disturbed nocturnal sleep—namely, reduced sleep efficiency, increased number of awakenings, and increased time spent awake after sleep onset—significantly correlated with several VEMP scores. Interestingly, we found that higher oVEMP scores inversely correlated with the percentage of REM sleep. Previous studies did not look for correlations between REM sleep and VEMP findings, but consistently reported an association between VEMP abnormalities and the presence of RBD (8, 10), notably even during prodromal stages (12, 13). Early neurodegeneration of REM sleep-regulating structures, including cholinergic and monoaminergic brainstem nuclei, notably the pontine sublaterodorsal tegmental nucleus (31), and a progressive reduction in REM sleep with PD progression (32) have been well-documented. Taken together, the association of VEMP abnormalities with decreased REM sleep and presence of RBD in PD suggests a parallel degeneration of vestibular and sleep-wake-regulating brainstem structures with PD progression.

Several limitations of the study need to be acknowledged. First, the included sample size was small, and our findings therefore need to be confirmed by larger studies. For the same reason, the differences in oVEMP latencies between PD patients with and without imbalance should be regarded as hypothesis-generating only. PSP patients experience devastating physical and neuropsychological symptoms, which made the prospective inclusion and organization of the study examinations very challenging, and greatly depended on the continuous help of their caregivers. Therefore, the dropout rate in the PSP group was very high. However, as there were no demographic and clinical differences between included and dropped-out PSP patients, we believe that the obtained VEMP findings are still representative for this disease and not substantially confounded by an inclusion bias. Second, the mean age of PSP patients was higher than of PD patients. Due to age-related changes in VEMP signals, this might have contributed to the higher VEMP scores in the PSP cohort. However, as none of the VEMP parameters showed any correlation with age, we believe that the lack of perfect age matching between our PSP and PD patients should not be regarded as a significant confounder. Moreover, a study of 314 participants observed decreasing cVEMP amplitudes with age, but no effect of aging on cVEMP latency (33). Third, reliable oVEMP and cVEMP examinations require adequate patient cooperation, and excessive sternocleidomastoid muscle contraction or incomplete up-gaze may compromise the quality of cVEMP and oVEMP, respectively, especially in PSP patients. Although cVEMP were performed in each PSP patient according to recent international practice parameters (21), including monitoring of adequate sternocleidomastoid muscle contraction levels, and although none of the PSP patients had complete vertical ophthalmoplegia, with objective documentation of preserved up-gaze on EOG, we cannot exclude that reduced patient cooperation during VEMP testing might have contributed to the higher VEMP scores in PSP patients. Finally, we performed cVEMP testing by air-conducted sound, but did not exclude conductive hearing impairment by audiometry or tympanometry. Thus, we cannot reliably exclude that absent cVEMP signals were primarily caused by subclinical conductive hearing impairment. Indeed, bone-conducted vibration elicited normal cVEMP signals in 2 PD and 3 PSP patients, in whom air-conducted sound had failed to elicit any cVEMP signal. In 5 PSP patients, on the other hand, cVEMP signals could not be elicited by air-conducted sound nor bone-conducted vibration. Since a majority of patients had only cVEMP with air-conducted sound, and because cVEMP latencies obtained by sound and vibration may differ (34), we decided for the sake of methodological homogeneity to use only cVEMP findings tested with air-conducted sound.

In conclusion, the present study extends the emerging literature on ocular and cervical VEMPs in neurodegenerative disorders by showing that cervical and total VEMP scores are more pathological in PSP than in PD and that very high total VEMP scores occur only in PSP patients. This suggests that brainstem vestibular pathways are more severely damaged by tauopathy in PSP than by alpha-synucleinopathy in PD. It also encourages including VEMPs more frequently into the diagnostic armamentarium applied to patients with neurodegenerative Parkinsonian syndromes.

## Data Availability Statement

The raw data supporting the conclusions of this article will be made available by the authors, without undue reservation.

## Ethics Statement

The studies involving human participants were reviewed and approved by The Ethics Committee of the Canton of Zurich approved the study protocol (KEK-ZH-Nr 2017-01323). All patients gave written consent prior to study inclusion. The study was carried out in accordance with the Declaration of Helsinki. The patients/participants provided their written informed consent to participate in this study.

## Author Contributions

SC, PV, KW, DW, and YV conceived and designed the study. SC, PV, DW, EB, EW, CJB, and YV collected and analyzed the data. SC, PV, KW, and YV participated in drafting the article for important intellectual content. CRB, DS, and EW critically revised the manuscript and approved the submitted version. All authors contributed to the article and approved the submitted version.

## Conflict of Interest

The authors declare that the research was conducted in the absence of any commercial or financial relationships that could be construed as a potential conflict of interest.
