# Supplementary material for: Distinct Vestibular Evoked Myogenic Potentials in Patients With Parkinson Disease and Progressive Supranuclear Palsy
Source: Front Neurol. 2021 Feb 12;11:598763. doi: 10.3389/fneur.2020.598763 (PMC7906978; doi:10.3389/fneur.2020.598763)
Supplement: Supplementary file 1 [file Table_1.docx]

**Supplemental Table 1** Left-right comparison of oVEMP and cVEMP latencies in 10 PD patients with asymmetric motor disability.

**latencies (left) latencies (right) *p***

***PD patients with more prounced***

***motor disability on the left (n=6)***

oVEMP: n10 13.1 (13.0 – 13.3) 13.2 (12.8 – 13.9) 0.60

oVEMP: p15 16.5 (16.1 – 17.0) 17.9 (16.0 – 19.1) 0.22

cVEMP: p13 14.1 (14.0 – 14.8) 14.0 (13.4 – 15.3) 0.97

cVEMP: n23 22.9 (22.4 – 23.4) 23.2 (22.1 – 23.9) 0.84

***PD patients with more prounced***

***motor disability on the right (n=4)***

oVEMP: n10 13.1 (12.6 – 14.7) 13.1 (12.8 – 14.4) 0.93

oVEMP: p15 17.8 (16.4 – 19.3) 18.8 (16.7 – 19.7) 0.65

cVEMP: p13 13.3 (12.8 – 14.0) 14.1 (14.0 – 14.5) 0.08

cVEMP: n23 21.4 (20.9 – 22.3) 22.0 (20.6 – 24.3) 0.49

*Results are presented as median (25-75% interquartile range)*
